# A systematic workflow for utilizing and evaluating large language models in public health policy framing analysis

**DOI:** 10.1186/s13690-026-02089-6

**Published:** 2026-10-17

**Authors:** Johanna Wenderoth, Peter von Philipsborn, Anna Leibinger, Philipp Cimiano

**Affiliations:** 1 Chair of Public Health and Health Services Research, IBE, LMU Medizin, LMU Munich, Munich, Germany; 2 Pettenkofer School of Public Health, Munich, Germany; 3 Chair of Public Health Nutrition, University of Bayreuth, Bayreuth, Germany; 4 Cognitive Interaction Technology Center (CITEC), Bielefeld University, Bielefeld, Germany

**Keywords:** Framing analysis, Public health policies, Media analysis, Germany, Large language models, Tax on animal-based foods, Public health nutrition

## Abstract

**Background:**

For public health policy research, it is crucial to monitor policy debates to inform evidence-based policymaking. One popular method is to analyze the news for salient frames used to argue for or against a controversial topic. However, this method is time and resource-intensive since domain experts need to manually analyze relevant articles to identify frames. This study presents an approach for using large language models (LLMs) to extract frames and to systematically evaluate results.

**Methods:**

Building on the results of a framing analysis of the German media debate about a tax on animal-based foods, we developed a systematic workflow to utilize LLMs to extract all instances of the 10 most frequent manually coded negative frames from 46 news articles. With one prompt per article in a few-shot setting we generated a list of quote/frame pairs. We then evaluated results against human-coded ones and calculated precision, recall, and F1-score.

**Results:**

GPT-5 achieved 79.8% precision and 79.8% recall, identifying 75.2% of manually coded frames. Extracted quotes generally aligned with human-extracted text sections. Gemini 3.1 Pro Preview yielded higher precision but lower recall, resulting in a comparable F1-score (79.0% vs. 79.8% with GPT-5). These findings suggest the approach is generalizable across different frontier models.

**Conclusions:**

This study introduces a workflow for LLM-assisted frame extraction and transparent evaluation against an expert reference standard. Integrating this approach into public health policy coding can save resources and accelerate analysis. Ultimately, this could increase the representativeness and timeliness of data, enhancing our understanding of ongoing public health policy debates.

**Supplementary Information:**

The online version contains supplementary material available at https://doi.org/10.1186/s13690-026-02089-6.

**Table:** 

Text box 1. Contributions to the literature
• Framing analysis is used to study policy debates but relies on time-intensive manual coding, which can delay insights for policy making. The potential of LLMs to support framing analysis in public health debates has not yet been systematically evaluated.
• We present a workflow on how to use LLMs to identify policy frames on a tax on animal-based foods in German news coverage and validate results with frames identified by human coders.
• Our findings show that LLMs can assist researchers in analyzing media debates and in the future this approach could be incorporated into framing analyses, enabling more timely insights for public health policy.

## Background

While public policies can help improve the health of a population, new health policy proposals are often controversially discussed by policy actors and the public [1]. To gain support of policymakers and the public, thus increasing the chances of successfully introducing new legislation, it is important to understand the public debate [2, 3]. One popular method to analyze policy debates is a framing analysis [4], which seeks to identify the patterns or “frames” the media and different policy actors use to argue for or against a topic, making some points or issues more prominent while omitting others [2]. Framing analyses usually employ a manual, qualitative coding process [1, 5], where domain experts study relevant articles to identify frames [5, 6]. These frames can either be pre-defined generic frames for a variety of domains or issue-specific and purpose-built for the topic at hand [7, 8].

Entman [9] formally defined framing as: “To frame is to select some aspects of a perceived reality and make them more salient in a communicating text” [9]. Framing is “selection and highlighting, and use of the highlighted elements to construct an argument about problems and their causation, evaluation and/or solution” [9]. Boydstun et al. define framing as “portraying an issue from one perspective to the necessary exclusion of alternative perspectives” and emphasize that “framing can have a significant influence on public attitudes toward important policy issues” [5]. Several studies in health policy research have focused on framing analyses, including meat tax [10–12], sugar tax [13], tobacco control [14], and spirits tax [15].

Koon et al. conducted a scoping review to analyze how frames can influence the health policy process, reviewing 52 framing analysis articles from 12 countries [1]. They concluded that framing research is “an important vehicle for understanding human behavior in the health policy process”, “ultimately leading to a deliberative mode of policy analysis”. The usage of framing in debates can be an important catalyst for policy change [1].

As with qualitative research in general, conducting a framing analysis is a time-consuming and labor-intensive task, which limits the amount and recency of data that can be included in a study [8, 16]. In addition, the identification of frames is a somewhat subjective task and may be influenced by the coder’s perspective and beliefs [1, 17]. Thus, if coding is done by more than one coder, this can capture different (but valid) interpretations in assigning a frame [17]. Moreover, there is a continuous stream of new content with potential new frames [5].

Consequently, several strands of research work have looked into ways of automating the process of identifying frames in text [5, 18, 19]; Ali and Hassan [20] present an overview of computational approaches used in the past. One frequently used set of frames and data in computational framing analysis is the Policy Frames Codebook (PFC) along with the Media Frames Corpus (MFC) [20, 21]. The PFC proposed by Boydstun et al. [5] consists of 14 policy frames and a 15th “other” frame; these are applied by Card et al. [17] in the MFC to manually annotate news reports.

With the emergence of LLMs like those powering ChatGPT, which can approach human-level performance on various tasks, a significant improvement to the state-of-the-art of Natural Language Processing has been realized [16, 22]. Researchers have quickly started to explore the use of LLMs to support qualitative research [16, 23]. Within the public health domain Liu et al. [24] use GPT-3.5-Turbo to classify the quality of health news along nine criteria. Kim et al. [25] rely on LLMs to detect risk promoting vs. health supporting sentiments from social media messages on Human Papillomavirus vaccination and tobacco products, comparing results to human annotations. Similarly, Espinosa and Salathé [26] use multiple LLMs to classify the stance towards vaccination in social media posts. Harris et al. [27] assess the usage of LLMs in a range of Public Health classification and extraction tasks.

For framing analyses, the use of LLMs has mainly been explored in the social sciences. The approaches can be broadly divided in using generic vs. issue-specific frames and assigning a single frame or multiple frames per article in a multi-label setting (the common approach in qualitative framing analysis) [28]. Kuang et al. [8] explore using BERT to identify seven generic media frames, allowing for multiple frames per article on the topic of the Russia-Ukraine war. Arora et al. [28] conduct a multi-label framing analysis of U.S. based news articles using Mistral-7B to extract generic and issue-specific frames. Kaur and Arora [21] use Mistral-7b to extract a list of 14 generic frames from the MFC along with reasoning for the classification. Separately, they prompt Command-R, an LLM, to extract a set list of 17 issue-specific frames related to war and peace along with a text excerpt and reasoning.

A lot of research in automatic frame classification so far has focused on the task of classifying frames on headline or article level with a single frame [6, 16]. The application of LLM prompting for framing extraction in health policy research has been sparse. Barrio et al. [6] classified the headlines of articles related to the COVID-19 anti-vaccine movement into five news frames, using GPT-3.5. Similar to the PFC frames, these five frames are broad in definition, so their usage makes it hard to capture nuances of a text [20], which in policy research is often necessary. In contrast, issue-specific frames allow for a more fine-grained analysis of a particular issue [28]. To summarize, to our knowledge, in the health policy area, there has been little research on automatic frame extraction using LLM prompting, especially of issue-specific frames and of multiple frames per document with a quantitative evaluation against domain-expert created results.

With this study, we aim to fill this gap and present an approach how to use LLMs to automatically (1) extract (multiple) quotes from articles, (2) classify these into issue-specific frames and (3) systematically assess the quality of the results. Quotes were extracted from articles used in a qualitative framing analysis on the German media debate around taxation of animal-based foods [29]. Previously, a domain expert had reviewed news articles and extracted relevant quotes, assigning them to issue-specific frames. We used these manual results by the domain expert to benchmark the LLM’s automatically created results. Since arguments against a policy are of particular interest to policymakers and as a better understanding of these can help public policy advocates to develop strategies countering these arguments [2], our study focused on extraction of quotes and frames where a tax on animal-based products was framed negatively. Our goal was to develop a workflow on how to use LLMs to assist a qualitative framing analysis and to rigorously and transparently evaluate the quality of results produced by the LLMs.

## Methods

The following section provides an overview of the systematic workflow we developed to automatically extract quotes from news articles, classify them into frames, benchmark the results against human results, and calculate widely used performance metrics such as Precision, Recall and F1-score.

### Input data

The data used as input for the LLM was first collected as part of a qualitative framing analysis [29]. The aim of this study was to understand how taxes and levies on animal-based foods have been framed in the German media [29], as such taxes and levies could serve as an important public health lever for a shift towards a healthier and more sustainable diet. Specifically, the data studied as part of the framing analysis consisted of 381 news articles from 10 national German newspapers and eight trade press outlets discussing an increase in price of animal-based foods through taxes and published between February 2019 and July 2023 [29]. All articles were written in German.

A domain expert manually identified 2700 relevant quotes from the articles and coded them for frames. The underlying codebook and frame boundaries were developed through an iterative, consensus-based approach between all authors [29]. For the purpose of quality control, a second author checked 30% of all coded text segments [29]. Differences in understanding were discussed and resolved, and refinements to the codes and codebook performed [29]. This ensured that the frames reflected a collective expert interpretation of the German media landscape, rather than a single individual’s bias.

We used the coded quote/frame pairs from the previous study [29] and the 381 articles as a starting point for our study.

### Process and methodology

**Fig. 1:**
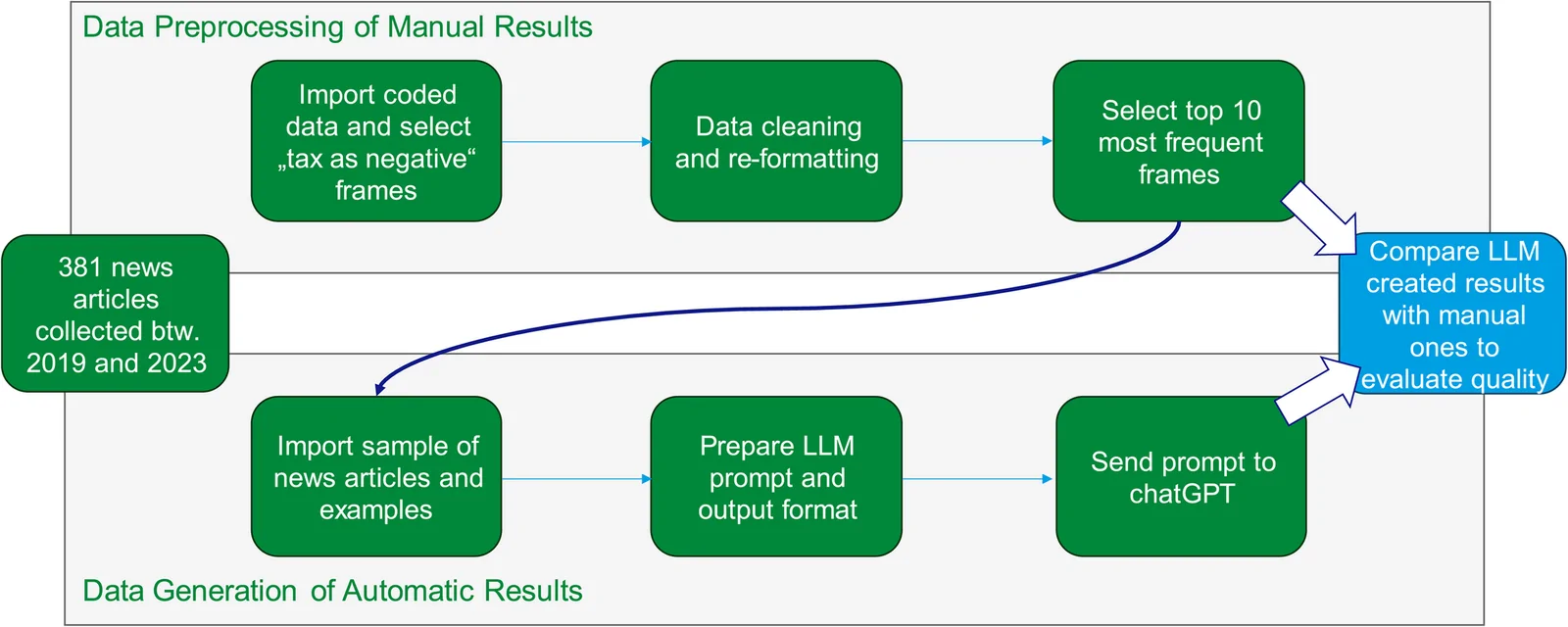
Process overview of data preprocessing of manual results, data generation of automatic results and evaluation

Figure 1 shows the workflow we have developed to generate automatic quote/frame pairs and to assess the quality of automatic results compared to manual results. All steps were implemented as Python programs (version 3.11) [30], except for the evaluation of additional LLM-created frames, which was performed in Microsoft Excel.

#### Data preprocessing steps of manual results

We first imported the manually extracted quote/frame pairs into Python and filtered them to those containing “tax as negative” frames. As the most frequently appearing frames are the most important frames from a policy making perspective, we restricted the LLM task to only identify and extract quote/frame pairs fitting into one of the top 10 “tax as negative” frames. These ten “tax as negative” frames accounted for 47% of all coded quotes and are the following: (1) tax as amplifying inequalities, (2) tax money as not going to stated aims, (3) tax as not improving animal welfare, (4) regulations and control of agricultural practices needed, (5) tax as hurting domestic production, (6) subsidies need to be reformed, (7) tax as hurting better products disproportionately, (8) earmarking as not possible, (9) higher taxes as politically not feasible, (10) other sources of funding possible. A description of these frames can be found in additional file 1, Sect. 3.

#### Data generation steps of automatic results

In order to adapt the LLMs to the task at hand, we relied on in-context learning based on 20 examples sampled from 18 articles as training data in a so-called few-shot setting (see additional file 1, Sect. 4). Of the 202 articles that contain one of the top 10 negative frames, we excluded the 18 articles that we used for training the LLM. Of the 184 articles left, we took a random sample of 25%, i.e. 46 articles, that we used for this study. The average length of these articles was 6763 characters or 917 words.

To build the prompt, that is the instruction defining the task for the LLM, we utilized the open source LangChain Framework [31, 32]. Our prompt structure followed the LangChain guide [33] to extract structured information from text and consisted of four parts: (1) a system message to give background and general instructions on the task, (2) a description of the desired output format of the response (quote/frame pairs), (3) the 20 examples, and (4) the text of the news article from which the quotes and framings should be extracted. Next, our script looped over all articles sending 46 requests to ChatGPT (model GPT-5) and storing the returned quote/frame pairs for evaluation.

Based on the quality of results retrieved, we iteratively improved the prompt and followed tactics in the Prompt engineering guide provided by OpenAI [34]. The complete prompt can be found in additional file 1, Sect. 1.

### Evaluation of results

In order to compare manual and automatic results, we had to evaluate whether an automatic result was correct. Each result was either (1) a matching frame which had also been found manually or (2) an additional frame, which had not been identified during manual coding. The first case was straightforward: our script classified the result to be correct, as the same frame was identified manually. For matching frames, we also looked at how often the quote matched as well, as a quote match was direct evidence for the frame classification to be grounded in the text rather than a result of chance agreement by the LLM.

To compare quotes, we developed a heuristic: Quotes were determined to be equivalent, i.e. from the same text segment within the article, if at least one of the following three criteria applied: (1) the two quotes are more than 70% alike based on a similarity ratio [35], (2) the LLM found a quote that was a subset or superset of the manual quote or, (3) the two quotes had at least one sentence in common.

For the second case, that is, the additional frames not identified during manual coding, JW evaluated the frame to be either “fitting” or non-fitting by judging each quote/frame pair. A second author, AL, also independently evaluated the frames and any differences were discussed to reach consensus. An additional frame was classified as fitting if it made sense considering the information provided to the LLM (i.e. the description of the task, the names of the ten frames and the twenty examples). As an additional indication on the appropriateness of the extracted additional frame, using the above described heuristics, we flagged whether the same text segment the quote originated from had also been coded by the domain expert but classified into a different (but related) frame (as the domain expert coded from 54 different ‘tax as negative’ frames).

#### Overall frequencies and agreement

To be able to judge the quality of the automatic results and compare results between varying prompts, we computed overall frequencies, comparing the total number of frames extracted by the LLM with the number of manually assigned frames.

To better understand the agreement between manual and automatic results, we calculated the proportion of manually identified frames that were also found by the LLM. For these matching frames, we then calculated the proportion of matching quotes.

#### Frequencies by frame and additional frames analysis

Next, we analyzed the frequency of each frame in order to determine for which frames the LLM had extracted more or less instances than the domain expert and how many results were correct. This helped to compare the performance of the LLM across different frames. We also took a detailed look at the additional frames discovered by the LLM, categorized them as fitting or non-fitting, and identified patterns that helped explain the differences between manual and automatic results.

#### Quality metrics precision, recall and F1-score

We used the standard information retrieval metrics precision, recall, and F1-score [36] to compare human to LLM results.

Table 1 shows the definition of these metrics. Increasing precision is equivalent to reducing false positives, which, in our case, translated to fewer cases where the LLM assigned incorrect frames to an article. Increasing recall reduces the number of false negatives, which, in our case, was equivalent to extracting more relevant frames. We also calculated the F1-score as the harmonic mean between precision and recall. The F1-score is low when precision and/or recall are low, and high when both metrics are high. Attempting to balance precision and recall, we judged one run to be better than the other if its F1-score was higher.

**Table 1:** Definition of metrics used [36, 37]

Metric	Standard Formula tp = true positives, fp = false positives, fn = false negatives	Explanation
Precision *P*	$\:\frac{tp}{(tp+fp)}$	The proportion of all frames found which are correct/fitting
Recall *R*	$\:\frac{tp}{(tp+fn)}$	The proportion of all correct/fitting frames found
F1-score	$\:\frac{2PR}{(P+R)}$	Single measure which trades off precision and recall

We calculated precision as the number of correct/fitting frames found divided by all frames extracted. Because the LLM extracted additional frames that were not coded during the manual coding process for the same article, we defined recall as the set of correct results encompassing all human results plus the additionally verified results identified by the LLM. While even this recall figure was likely to miss some quote/frame pairs that another LLM or human coder would have found, we decided to approximate the true recall in this way rather than evaluating recall only with respect to the original manual results, as this would have underestimated the actual recall.

Our script calculated precision, recall, and F1-score across all articles, giving a higher weight to articles with more frames.

### Sensitivity analysis

To understand the influence that different settings have on the result, we also conducted a sensitivity analysis. In particular, we conducted experiments with two alternative settings to the ‘standard’ run described so far. In one experiment we reduced the number of examples provided to the LLM from two examples per frame to only one example per frame (10 examples in total), in another we used a German instead of an English prompt.

In addition, to better understand the robustness and reproducibility of our method, we tested running our extraction and classification task with Gemini 3.1 Pro Preview; to analyze how our method, developed and tested using ChatGPT, would perform using an entirely different frontier model. Like our other runs as part of the sensitivity analysis, we left everything else (the prompt and all other settings) unchanged.

### Accounting for variance in automatic results

Even for identical prompts the resulting output varied for every run (+/- 3% in number of results and +/- 1.5% points (pp) in F1-score). To reduce randomness, we performed three consecutive repetitions for both the ‘standard’ run and all sensitivity analyses, reporting the results from the run that achieved the median F1-score.

## Results

### Frequencies

#### Overall frequencies and agreement

**Fig. 2:**
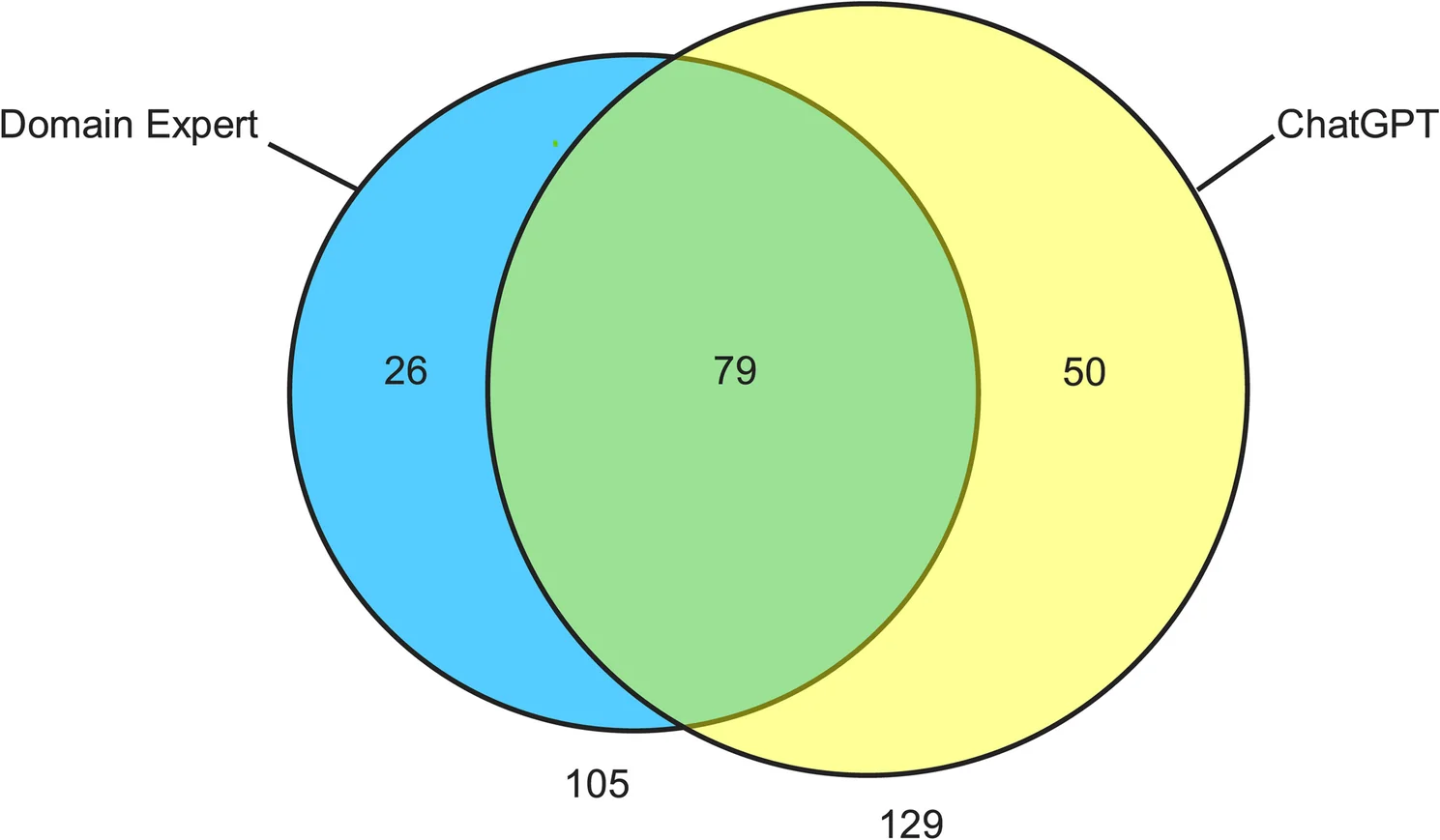
Total frames found by domain expert and ChatGPT and overlapping results

Figure 2 provides an overview of the total frames found and the agreement between manual and automatic results using GPT-5. Overall, the LLM extracted 129 quote/frame pairs; 22.9% more than were extracted manually by the domain expert. Of the 105 frames identified manually, the LLM extracted 79 (75.2%) as well, i.e. the same frame for the same article. In 94.9% of the cases, the quote in addition to the frame also matched.

#### Frequencies by frame and additional frames analysis

Table 2 provides a detailed breakdown of frequencies by frame. There were a total of 50 additional frames for which ChatGPT extracted a quote and classified it into one of the top 10 frames, but the same frame was not identified by the manual coder for the same article. After manually reviewing the 50 additional quote/frame pairs, we identified 48.0% as fitting, given the information that the LLM was provided with (i.e. the top 10 frames with two examples each). Looking at the results per frame, the proportion of additional fitting frames ranges from 0% to 100%, with all additional frames found for “Amplifying inequalities” not fitting, while for the frames “Hurting better products” and “Earmarking not possible“, all additional frames were fitting. 

**Table 2:** Comparison of automatic and manual results by frame

Frame	automatic results	manual results	frame matches	% manual frames found	additional frames found	% additional frames fitting
Regulations needed	21	13	11	84.6%	10	50.0%
Hurting domestic production	12	12	9	75.0%	3	33.3%
Other funding possible	14	9	5	55.6%	9	22.2%
Subsidies need reform	10	11	7	63.6%	3	66.7%
Hurting better products	10	11	8	72.7%	2	100.0%
Earmarking not possible	9	7	4	57.1%	5	100.0%
Amplifying inequalities	17	14	12	85.7%	5	0%
Not improving animal welfare	17	10	10	100.0%	7	57.1%
Not going to stated aims	9	10	6	60.0%	3	33.3%
Not politically feasible	10	8	7	87.5%	3	66.7%
**Overall**	**129**	**105**	**79**	**75.2%**	**50**	**48.0%**

For nearly half (24, 48.0%) of additional frames, the human coder had also extracted a quote from the same text segment but coded this into a different frame. In five cases, the human coder assigned a different top 10 frame to the quote. In the remaining 19 cases, the manually assigned frame was not among the top 10 most frequent frames we asked ChatGPT to use. In many cases, the manual and LLM extracted frames were conceptually related: e.g. “Tax as causing legal problems: Earmarking as not possible” vs. “Tax as causing legal problems: Earmarking as against EU law”. As ChatGPT solely learned from examples, without a clear description of or distinction between frames, such frame assignments were judged as fitting.

There was one instance where ChatGPT hallucinated, as the quote returned did not exist in the article. Other reasons for the additional frames found were that ChatGPT assigned an overly broad frame, misunderstood the frame definition, or identified an additional quote the domain expert had overlooked. The following is an example of a quote ChatGPT extracted and assigned to the frame “Other sources of funding possible”: “‘We therefore need a state animal welfare premium’, demands the industry representative. After all, it must remain possible for the majority of consumers to be able to afford meat.” An animal welfare premium is a kind of tax, so this is not a different source of funding and not an argument against introduction of a tax.

### Quality metrics precision, recall and F1-score

#### Overall figures

Table 3 shows the results for overall precision, recall, and F1-score. Of all results extracted, 79.8% were correct, and 79.8% of all correct results have been found, combining to an F1-score of 79.8%. While ChatGPT generally extracted more quote/frame pairs than the human coder, this came at the expense of a lower precision (79.8% vs. ~100% assumed precision for manual results).

**Table 3:** Overall precision, recall, and F1-score

Metric	Automatic
precision	79.8%
recall	79.8%
F1-score	79.8%

#### Sensitivity analysis

To understand the influence different settings have on the model’s performance, we present the results of different runs in Table 4. The run variant with only 10 examples as context delivered the most results (143) and identified the highest proportion of manual frames out of all runs (78.1%) but yielded the lowest precision. The prompt in German reaches slightly higher results than the standard run with an F1-score of 80.3% (+ 0.5 pp).

Running our prompt using Gemini 3.1 Pro Preview delivered the lowest number of results (93) and identified 67.6% of all manual frames, 7.6 pp below GPT-5. It delivered by far the highest precision (89.2%) of all runs (+ 9.4 pp vs. GPT-5).

**Table 4:** Results comparing runs with different settings

Run variant	# total results	% of manual frames found	% where quote matched in addition to frame	Overall precision	Overall recall	Overall F1-score
Standard Prompt, GPT-5	129	75.2%	94.9%	79.8%	79.8%	79.8%
Prompt with 10 instead of 20 examples, GPT-5	143	78.1%	93.9%	72.7%	81.9%	77.0%
Prompt in German, GPT-5	126	75.2%	93.7%	81.0%	79.7%	80.3%
Standard Prompt, Gemini 3.1 Pro Preview	93	67.6%	97.2%	89.2%	70.9%	79.0%

## Discussion

This study introduced an approach based on LLM prompting for framing analyses in the Public Health domain. Specifically, we created a systematic workflow to utilize LLMs to extract multiple relevant quotes from news articles discussing a tax on animal-based foods and to classify these into a set of frames, with one prompt per article. The approach includes rigorous and transparent evaluation of the LLM-created results against the results of a domain expert. As the results of this study show a promising level of accuracy, it provides a proof-of-concept for the feasibility of using LLMs to support researchers in conducting framing analysis. This approach, which works across different frontier models, could be incorporated into future framing analysis methodologies.

### Summary of main results

Using LLM prompting with GPT-5 to extract quotes from articles and to classify them into a set of frames, ChatGPT was able to extract 75.2% of all frames identified manually and furthermore discovered 50 additional results, of which 48.0% were correct, resulting in an overall recall of 79.8%, precision of 79.8% and the F1-score of 79.8%. Cross-model testing showed that Gemini 3.1 Pro Preview produced a different performance profile to the results achieved with GPT-5, with much higher precision but lower recall. However, the F1-score remained comparable (79.0% vs. 79.8% with GPT-5), proving the method’s reliability across different frontier AI architectures.

### Interpretation

The presented LLM-assisted approach was able to identify the majority of frames the domain expert had found and at high precision. While the LLM did not reach the precision of a domain expert, not always “grasping” the meaning assigned to the frames by the researcher or not considering the wider context of the quote (e.g. if the argument actually pertained to the introduction of an animal-based food tax or not), the LLM helped discover additional results: Both the human coder and ChatGPT identified frames missed by the other one. The presented workflow can be used to complement a human coder and can improve the overall results of a framing analysis. In general, the presented LLM prompting method is a flexible approach which can be configured to prefer higher recall – so helping to discover new arguments – or higher precision for high confidence in results. The specific use case depends on resources available and whether the goal is to e.g. find all frames in existing articles or quickly get an idea of trends existing in new, unseen articles whilst limiting the amount of text the human coder needs to verify.

### Comparison to similar studies

Comparing the results of this study to previous research is difficult, mainly because our methodology in which we combine the two tasks of quote extraction and subsequent frame classification differs from previous research efforts towards the automatic identification of arguments. In addition, we use a novel data corpus with issue-specific frames not used in the past. Nevertheless, we can look at results of related research efforts. Barrio et al. classify headlines into five generic frames and correctly identify 72% using GPT-3.5 [6]. Kuang et al. [8] use LLM BERT to identify seven generic media frames in a multi-label setting and get 75.1% of human annotations predicted correctly. With GPT-5 we correctly identify 75.2% of all manual frames, even though our method includes the task of quote extraction preceding the classification task, where all erroneously extracted quotes will, per definition, be misclassified.

Arora et al. [28] use Mistral-7B to extract 15 generic frames from the MFC in a multi-label setting and achieve an F1-score of 0.5. Naderi and Hirst [18] use recurrent neural network with word embeddings to classify sentences into one of the top 5 Media Frames and reached an F1-score of 70.7%, below our F1-score of 79.8% reached with GPT-5.

### Strengths and added value of our study

Our work differs from past work in policy research in several major aspects. Firstly, we are using LLM prompting to tackle two tasks in one: data extraction and data classification, making the task more complex, as it involves more steps but also enabling us to discover new quote/frame pairs. With this approach, we are able to reproduce the steps that a domain expert would undertake to read an article, identify relevant quotes and classify those into frames.

Secondly, this approach can serve as a systematic workflow for using LLM prompting to extract quote/frame pairs from text and then benchmark the results with manual results, applying well-established quality metrics. Since we are extracting frames in a multi-label setting, a variety of frames can be extracted even from complex articles containing diverse arguments. An aspect to consider as well is how much time can be saved using this LLM-assisted approach. Using GPT-5, the LLM had identified 75% of all human frames (True positives), but in addition, 20.2% of frames identified by the LLM were false positives (Gemini 3.1 Pro Preview: 10.8%). These false positives need to be filtered. If we assume the time required for a domain expert to validate a frame to be approx. 60–90 s, with an average of 3 frames to evaluate per article, this is significantly lower than the time required to manually read and code an entire article from scratch (20–40 min). Thus, the use of LLMs in framing analysis can be net-positive, as the time savings already outweigh the additional cost of human-in-the-loop validation. The LLM can be useful as a second coder and can help to confirm manually extracted frames or to discover additional frames.

Finally, we demonstrated our method’s capabilities using news articles in German language with a set of 10 issue-specific frames. As the frames and domain expert results were not publicly available as training data, there is no possibility the LLM was trained on the data and just recollected the correct results.

### Limitations

Despite these promising results, there are some limitations to this study. Since we chose to only extract the top 10 frames of the 54 original frames, we only presented ChatGPT with these top 10 frames for classification. As a result, the LLM categorized some quotes in one of the top 10 frames, while one of the omitted frames would have been more fitting. In addition, ChatGPT was provided with two examples per frame but no general descriptions of a frame. These decisions lead the LLM to use less differentiated information on the task than the domain expert used, making this not a perfect like-for-like comparison. In addition, the LLM would only be able to identify previously defined frames in new articles, not potentially newly emerging frames. Also, the used top 10 frames only accounted for 47% of human-coded “tax as negative” frame instances. In an exploratory post-hoc analysis with a more complex set of 20 frames (representing 77% of human-coded instances), GPT-5 identified 66.7% of the human frames, an 8.5 pp reduction from the baseline. This indicates that as frame frequency decreases and prompt complexity increases, model recall declines. Consequently, while the approach is highly effective for dominant discourses, further improvements to the model (such as providing explicit frame definitions within the prompt or batching frames into separate prompts) are required before this approach can reliably detect less frequent arguments.

Another limitation is the somewhat subjective nature of qualitative research, which affects the obtainable accuracy from the model, as differences in results can be expected in frame classification, even between humans.

To ensure our approach still works as intended over time, ongoing quality testing and maintenance (and potentially adjustments to the prompt) will be necessary.

## Conclusions

With this study we were able to show that LLM prompting can be used for the specialized task of data extraction and classification in health policy framing analysis and achieve results comparable to approaches that only consider one of the two tasks (quote extraction or classification). We have developed and empirically validated a novel method of how to accomplish the task of extracting and classifying quotes end-to-end, starting with a set of articles and frames to retrieve automatic results and to systematically quality check these against human results with standard metrics.

We can apply the presented systematic workflow to (1) use the LLM to serve as a second coder on the same articles or (2) point to classifications of the manual coder which might need review, and (3) to extract frames from new, unseen articles or other material in a fast and scalable way. The additional insights gained can further broaden our understanding of the media debate, maintain recency of information and discover trends like shifts in public opinion.

Our results, however, also highlight challenges, as nuances in the human interpretation of natural text based on context are still challenging for a state-of-the-art LLM to capture in full. The LLM does not reach the quality of a human coder and cannot replace human coding but can improve the overall results of the framing analysis if used in addition.

Further research should include (1) using further background knowledge by including frame descriptions to the prompt, (2) splitting the two tasks into two separate prompts, (3) expanding beyond the top 10 frames, and (4) extracting other information like policy actors.

## Supplementary Information

Supplementary Material 1.

## Data Availability

The coded quotes and frames as part of the framing analysis which served as input to this study are available at OSF: https://osf.io/5ntvq/. The list of newspaper articles which were used under license for the current study can be found in additional file 1, Sect. 2. The Python code written for automated text extraction and classification is permanently archived on Zenodo (https://doi.org/10.5281/zenodo.23351659) and is available under the MIT License.

## Abbreviations

LLM: large language model
pp: percentage points
PFC: Policy Frames Codebook
MFC: Media Frames Corpus
