# Supplementary material for: A systematic workflow for utilizing and evaluating large language models in public health policy framing analysis
Source: Arch Public Health. 2026 Oct 17;84:225. doi: 10.1186/s13690-026-02089-6 (PMC13679237; doi:10.1186/s13690-026-02089-6)
Supplement: Supplementary file 1 [file 13690_2026_2089_MOESM1_ESM.docx]

Additional file 1

Supplementary material to: Wenderoth, J., von Philipsborn, P., Leibinger, A., Cimiano, Philipp. *A systematic workflow for utilizing and evaluating large language models in public health policy framing analysis.* Manuscript submitted to Archives of Public Health, 2026.

Table of Contents

[1. Overview of prompt implementation	2](#_Toc230021879)

[2. List of articles used in extraction of automatic results	4](#_Toc230021880)

[3. Description of top 10 frames	7](#_Toc230021881)

[4. 10 manual extracted examples used in few shot prompt	8](#_Toc230021882)

[5. Example of extracted quotes for the same frame	11](#_Toc230021883)

[References	12](#_Toc230021884)

# 1. Overview of prompt implementation

**System message***: "* *I need your help as a Public Health Nutrition expert! Imagine you've been following the German media debate about a potential tax on animal-based products like milk, meat, and eggs. I'll provide you with a complete article, and your job is to find arguments against the tax and alternative solutions proposed.*

*Here's what I need you to do:*

*1. Find relevant sections: Search the article for text segments that discuss a potential tax, levy, or animal welfare surcharge on animal-based products in Germany. Look for keywords like "Mehrwertsteuer" (VAT), "Steuer" (tax), "Verbrauchsteuer" (excise tax), "Tierwohlcent" (animal welfare cent), "Tierwohlabgabe" (animal welfare levy), "Fleischsteuer" (meat tax), or "Borchert-Kommission" (Borchert Commission).*

*2. Filter for criticism: From these sections, identify parts which express criticism of such a tax. These "policy actors" might explain why they dislike the tax or suggest better alternatives. Discard sections which voice an opinion clearly in favor of such a tax.*

*3. Extract arguments: See if the criticism falls into one of the 10 argument categories I'll provide (with examples). If it does, extract the category along with a 2-4 sentence quote from the article in the CategoryQuote_pair format. If there's no relevant criticism or the sequence is not about a tax or levy on animal-based foods, don't respond."*

**Output schema**, we called “*CategoryQuote_pair*”. This consists of two attributes:, ‘Category’ and ‘Quotation’. We instruct the LLM to extract a list of frame category/quote pairs for an article, but allow not to return anything if it does not fit these categories, to avoid the model from making up answers.

- Category description*: “The matching category for the argument. One of the following:*
  - *Tax as hurting consumers: Tax as amplifying inequalities*
  - *Tax as not achieving the declared intentions: Tax money as not going to stated aims*
  - *Tax as not achieving the declared intentions: Tax as not improving animal welfare*
  - *Tax as the wrong instrument: Regulations and control of agricultural practices needed*
  - *Tax as causing unintended disruption (market/industry/farmers): Tax as hurting domestic production*
  - *Tax as the wrong instrument: Subsidies need to be reformed*
  - *Tax as causing unintended disruption (market/industry/farmers): Tax as hurting better products disproportionately*
  - *Tax as causing legal problems: Earmarking as not possible*
  - *Tax as the wrong instrument: Other sources of funding possible*
  - *Higher taxes as not wanted by anyone: Higher taxes as politically not feasible”*
- Quotation description*: “Exact quote from the text which supports the argument.”*

**Examples:** We use few-shot prompting, by including a list of examples as part of the prompt. Each example contains a. larger text segment from the article (as an example for input) along with the matching category and citation pair the LLM should deliver as output. To keep the examples short, in contrast to the actual request, we only provide a larger section around the quote as context instead of the whole article. This serves as training data for the LLM for it to learn the task. We are including two examples for each of the 10 categories.

Here is one reference example to illustrate (translated to English):

Example input:

“The tax proponents from the agricultural committee faced even more headwinds, some of which were actually worth considering. For example, an exception would have to be guaranteed for organic meat. This is already drastically more expensive than conventionally produced meat, and a VAT increase would push its price up even more sharply than that of cheap meat. This would absurdly penalize the better treatment of plants, resources, and animals. If only the VAT were adjusted as proposed, the price increases would probably be too small to have a noticeable impact on meat consumption."

Example output (following the *CategoryQuote_pair schema)*

- Category=”Tax as causing unintended disruption (market/industry/farmers): Tax as hurting better products disproportionately”,
- Citation=”This is already drastically more expensive than conventionally produced meat, and a VAT increase would push its price up even more sharply than that of cheap meat. This would absurdly penalize the better treatment of plants, resources, and animals”

**Human input**: content of one complete article. The Articles range in length between ~490 and ~2400 words.

# 2. List of articles used in extraction of automatic results

- Allgemeine Fleischer Zeitung:2020-07-01 Kein Raum fuer Lockvogelpreise
- Allgemeine Fleischer Zeitung:2020-09-02 Ein Preis fuer gutes Gefuehl
- Allgemeine Fleischer Zeitung:2020-09-09 Fleischkonsum soll teurer werden
- Allgemeine Fleischer Zeitung:2021-03-24 Gefordert ist eine ehrliche Diskussion
- Agrarheute: 2021-09-10 Verpflichtender Tierwohl-Beitrag soll Borchert -Plan finanzieren
- Agrarzeitung: 2020-03-06 Peter Seeger zum Borchert-Papier - Ist die Reduktion der Tier- haltung das Ziel?
- Agrarzeitung: 2021-01-15 Bundestagswahl - Elefantenrunde zur Landwirtschaft
- Agrarzeitung: 2021-01-22 Aus zwei mach eins
- Agrarzeitung: 2021-01-24 Schweinehaltung - Schwarz-gruener Schulterschluss
- Agrarzeitung: 2022-06-08 Tierhaltungskennzeichnung - Oezdemir praesentiert
- Agra Europe: 2020-07-06 BDM und Freie Bauern sehen Tierwohlabgabe kritisch
- Frankfurter Allgemeine Zeitung: 2019-08-07 Jetzt melden sich die Gegner der Fleisch- Steuer
- Frankfurter Allgemeine Zeitung: 2019-08-13 Wer Fleisch isst muss kein schlechtes Ge- wissen haben
- Frankfurter Allgemeine Zeitung: 2021-05-29 Staendig lockt das Fleisch
- Frankfurter Allgemeine Zeitung: 2021-12-27 Keine Ramschpreise fuer Lebensmittel
- Focus: 2019-08-31 Von Alois Gerig CDU-Abgeordneter im Deutschen
- Focus: 2020-07-18 Eine Schweinerei kommt selten allein
- Fleischwirtschaft: 2020-05-01 Interview - Langfristig wird das Geld nicht reich
- Fleischwirtschaft: 2020-08-09 Interview - Wir brauchen zuallererst vernuenftige
- Fleischwirtschaft: 2021-08-20 Bundestagswahl 2021 Weniger industriell und global
- Lebensmittel Praxis: 2019-09-17 Auf in den Kampf
- Lebensmittel Praxis: 2021-04-16 Wer bezahlt das Tierwohl
- Lebensmittel Zeitung: 2019-08-07 Vorstoss von Gruenen und SPD - Hoehere Mehrwertsteuer auf Fleisch gefordert
- Lebensmittel Zeitung: 2020-01-10 Initiative Tierwohl stellt sich ab 2021 neu auf
- Lebensmittel Zeitung: 2021-04-06 PKW-Maut als abschreckendes Beispiel
- Spiegel: 2021-11-06 Wir haetten uns mehr kuemmern muessen
- Süddeutsche Zeitung: 2019-08-08 Kleine Zahlen mit grosser Wirkung
- Süddeutsche Zeitung: 2020-08-11 Preis der Moral
- Süddeutsche Zeitung: 2021-03-02 Die armen Schweine
- Top Agrar: 2020-04-17 Wir brauchen eine Annaeherung
- Top Agrar: 2023-06-20 Mit der Ampel koennen Bauern nicht zufrieden sein
- Der Tagesspiegel: 2020-05-19 Scheuer stellt sich vor die Schlachthoefe
- Der Tagesspiegel: 2020-07-04 Die Preisfrage in den Supermaerketen dominiert da
- Der Tagesspiegel: 2021-12-31 Abgaben auf Obst und Gemuese
- Der Tagesspiegel: 2022-06-08 Haltung zeigen
- Welt: 2019-08-11 Wie reagiert der Markt auf mehr Abgaben
- Welt: 2020-06-21 Mehr als 1300 Corona-Faelle in Fleischfabrik
- Welt: 2020-09-04 Verbraucher-Label sind wohlfeile Gewissensappel
- Welt: 2021-04-14 Das Gefluegel-Paradox
- Welt: 2021-12-30 Lebensmittelpreise - Greenpeace fordert hoehere
- Zeit: 2019-08-15 Jetzt die Taten
- Zeit: 2021-03-04 Neue Studie zur Nutztierhaltung
- Die Tageszeitung: 2019-12-18 Gans oder gar nicht
- Die Tageszeitung: 2020-02-04 Lobbyisten fuers Essen
- Die Tageszeitung: 2021-01-21 Bauern haben selbst Schuld
- Die Tageszeitung: 2022-09-12 FDP geraet wegen Tierwohlcent unter Druck

# 3. Description of top 10 frames

*Descriptions and frames developed in Leibinger et al. [1]; reproduced here for reader convenience*

| ***Frame*** | ***Description*** |
| --- | --- |
| **Tax as the wrong instrument: Other sources of funding possible** | These arguments explore funding options beyond taxation. |
| **Tax as the wrong instrument: Regulations and control of agricultural practices needed** | These arguments focus on the necessity of regulating and monitoring agricultural practices. This is often seen as more effective than taxes and subsidies. |
| **Tax as the wrong instrument: Subsidies need to be reformed** | These arguments usually refer to the fact that most agrarian subsidies come from the EU at the moment (through the CAP: Common Agricultural Policy) and are distributed to farmers based on hectare size of the farms. This is often criticized and it is suggested that other factors, such as sustainability or animal welfare should also be considered. |
| **Tax as causing legal problems: Earmarking as not possible** | This group of arguments points out that earmarking of taxes is not legally possible. In this group, it is either explicitly stated that this is according to German or EU law and regulation or it is not specified which laws are referred to. |
| **Tax as not achieving the declared intentions: Tax money as not going to stated aims** | These arguments suggest concern that the revenue generated from taxes is not effectively allocated towards their intended purposes. Instead, the funds may be lost or mismanaged within bureaucratic systems, leading to inefficiencies and diversion away from the original goals. |
| **Tax as not achieving the declared intentions: Tax as not improving animal welfare** | These arguments reflect the perception that taxes aimed at improving animal welfare do not directly lead to tangible improvements in animal welfare conditions. Despite the imposition of taxes or levies intended to enable investments in animal welfare, there might be obstacles that prevent an improvement of animal welfare. |
| **Higher taxes as not wanted by anyone: Higher taxes as politically not feasible** | These arguments discuss the notion that raising a tax on ABP may not be politically feasible as some politiciancs and parties are generally against it, which means that the required majorities in the govermnent can not be gained. In some cases it is stated that politicians do not want to be the ones to raise taxes, but in other cases no reasons are given. |
| **Tax as hurting consumers: Tax as amplifying inequalities** | These arguments reflect the view that taxation policies contribute to widening socioeconomic disparities by disproportionately disadvantaging certain groups. They suggest that increased taxes on ABP may exacerbate existing inequalities in economic inclusion and opportunity as a tax would not change dietary patterns of the rich, but exclude individuals living in low ses from making their own free choices about their diet. |
| **Tax as causing unintended disruption (market/industry/farmers): Tax as hurting domestic production** | These arguments claim that a tax that may only be applied to domestic products would thus disadvantage them and lead to more consumption of cheaper foreign products which in turn would make it harder for domestic producers to survive. |
| **Tax as causing unintended disruption (market/industry/farmers): Tax as hurting better products disproportionately** | These arguments suggest that especially a value added tax will disproportionately hurt higher-quality products as the tax premium depends on the price of the product. A value added tax will thus widen the price gap between cheaper and more expensive products. |

# 4. 10 manual extracted examples used in few shot prompt

*Frame and quotes developed in Leibinger et al. [1]; reproduced here for reader convenience*

| Article | Quote | Frame | Context of quote within article |
| --- | --- | --- | --- |
| AZ_2021-08-03_Studie - Fleischsteuer - Mehr Schaden als Nutzen | Der letzte Woche veröffentlichte Bericht über die nationale Ernährungsstrategie forderte eine Reduzierung des Fleischkonsums um 30 %, verzichtete aber auf eine Fleischsteuer, da dies "politisch unmöglich" sei. | Higher taxes as not wanted by anyone: Higher taxes as politically not feasible | Fleischsteuer ist ständig in der Diskussion Der letzte Woche veröffentlichte Bericht über die nationale Ernährungsstrategie forderte eine Reduzierung des Fleischkonsums um 30 %, verzichtete aber auf eine Fleischsteuer, da dies "politisch unmöglich" sei. |
| WELT_2019-08-08_Bundesregierung wiegelt bei Fleischsteuer ab | Das Finanzministerium stellte klar, dass bei Steuern eine Zweckbindung von Mehreinnahmen etwa für den Tierschutz nicht möglich sei. | Tax as causing legal problems: Earmarking as not possible | Die Bundesregierung und Spitzenpolitiker der Koalition halten wenig von einer Fleischsteuer, um den Fleischkonsum zu bremsen. Agrarministerin Julia Klöckner begrüßte am Mittwoch zwar, dass es eine Sensibilität dafür gebe, dass mehr Tierwohl auch mehr Geld koste. Die CDU-Politikerin fügte aber hinzu: "Das Geld muss nicht automatisch aus Steuererhöhungen kommen, sondern kann durch Schwerpunktsetzungen erreicht werden." Für die SPD lehnte ihr Parlamentarischer Geschäftsführer Carsten Schneider jede Diskussion darüber ab, die Mehrwertsteuer bei Fleisch von sieben auf 19 Prozent zu erhöhen. Agrarpolitiker von Grünen, SPD und CDU hatten dies ins Gespräch gebracht. Das Finanzministerium stellte klar, dass bei Steuern eine Zweckbindung von Mehreinnahmen etwa für den Tierschutz nicht möglich sei. |
| LP_2022-06-13_Vollkasko-Mentalitaet passe | Eine höhere Mehrwertsteuer hätte mit Sicherheit Nachteile für die Landwirte. Fleisch würde insgesamt teurer und der Unterschied zwischen preiswerterer Ware und Tierwohlprodukten würde sich erhöhen, weil der Aufschlag bei einem höheren Preis prozentual höher ausfällt. | Tax as causing unintended disruption (market/industry/farmers): Tax as hurting better products disproportionately | Wie soll Tierwohl finanziert werden? Konrad: Wir haben weder eine Abgabe noch eine Steuer auf Fleisch im Koalitionsvertrag festgeschrieben. Wir sind uns dessen bewusst, dass ein Weg gefunden werden muss, mehr Tierwohl finanzierbar zu machen. Eine höhere Mehrwertsteuer hätte mit Sicherheit Nachteile für die Landwirte. Fleisch würde insgesamt teurer und der Unterschied zwischen preiswerterer Ware und Tierwohlprodukten würde sich erhöhen, weil der Aufschlag bei einem höheren Preis prozentual höher ausfällt. |
| ZEIT_2020-02-11_Mehr Tierschutz kostet Geld | Würden wir die Abgabe nur auf deutsche Waren erheben, würden die Konsumenten verstärkt ausländische Lebensmittel kaufen und die Produktion würde sich ins Ausland verlagern. Davon hätten die Tiere nichts. | Tax as causing unintended disruption (market/industry/farmers): Tax as hurting domestic production | ZEIT ONLINE: Herr Grethe, das Kompetenznetzwerk plädiert für eine Abgabe, die in Zukunft auf Fleisch, Milch und Eier erhoben werden soll eine Art Steuer auf tierische Produkte. Wie soll die funktionieren?  Harald Grethe: [...] Erhoben würde die Abgabe dort, wo die Endverbraucher für Fleisch, Milch und Eier bezahlen, also im Supermarkt, in Gaststätten oder Kantinen. Es wäre eine Abgabe auf alle tierischen Produkte, unabhängig davon, ob diese in Deutschland oder im Ausland produziert worden sind. Würden wir die Abgabe nur auf deutsche Waren erheben, würden die Konsumenten verstärkt ausländische Lebensmittel kaufen und die Produktion würde sich ins Ausland verlagern. Davon hätten die Tiere nichts. |
| SZ_2019-08-23_Alles hat einen Preis | Die Reichen werden weiterhin ihr Fleisch essen, und die weniger betuchten Bevölkerungsteile sind wie immer die Leidtragenden. | Tax as hurting consumers: Tax as amplifying inequalities | Zum Nachteil für die Ärmeren Jetzt wird wieder über eine Erhöhung der Fleischpreise diskutiert, um angeblich das Tierwohl der zu schlachtenden Tiere zu verbessern. Über drei Ecken das Tierwohl zu verbessern kann nie funktionieren. Die Reichen werden weiterhin ihr Fleisch essen, und die weniger betuchten Bevölkerungsteile sind wie immer die Leidtragenden. |
| WELT_2019-08-28_Julia Kloeckner - Wir sehen eine Katastrophe be | Aber eine höhere Steuer auf Fleisch kommt nicht automatisch den Tierhaltern zugute, etwa für den Stallumbau. | Tax as not achieving the declared intentions: Tax as not improving animal welfare | WELT: Das meiste Fleisch in Deutschland ist sehr billig. Gibt es irgendeine Form von Abgabe oder Steuer zur Verteuerung von Fleisch, die für Sie denkbar wäre?  Klöckner: Mehr Tierwohl kostet mehr Geld, das gibt es nicht zum Nulltarif. Und die Mehrkosten werden die Landwirte allein nicht stemmen können. Auch wir Verbraucher sind gefragt. Wer 100 Gramm Hähnchenfleisch für 15 Cent anbietet oder kauft, der muss nicht von Tierwohl sprechen. Das ist unanständig.  Aber eine höhere Steuer auf Fleisch kommt nicht automatisch den Tierhaltern zugute, etwa für den Stallumbau. |
| SZ_2020-02-08_40 Cent mehr fuers Fleisch | Allerdings stößt die Idee auf breite Ablehnung, weil damit Konsumenten einseitig belastet werden und keinesfalls sicher ist, dass sich damit wirklich etwas ändert. Die Mehrwertsteuer fließt in den Bundeshaushalt und kann für alles Mögliche ausgegeben werden | Tax as not achieving the declared intentions: Tax money as not going to stated aims | Diskutiert wurde in den vergangenen Monaten auch über eine höhere Mehrwertsteuer von 19 Prozent für Fleisch- und Milchprodukte. Derzeit liegt der Satz bei sieben Prozent. Allerdings stößt die Idee auf breite Ablehnung, weil damit Konsumenten einseitig belastet werden und keinesfalls sicher ist, dass sich damit wirklich etwas ändert. Die Mehrwertsteuer fließt in den Bundeshaushalt und kann für alles Mögliche ausgegeben werden. Eine zweckgebunden Abgabe sollte nach Ansicht von Experten Landwirtschaft und Erzeugern zugute kommen, um etwa die Viehhaltung umwelt- und tiergerechter zu gestalten. |
| SPIEGEL_2021-11-24_Wer wo nachgeben musste und was jetzt kommt | Stattdessen sollen nun die »Marktteilnehmer« ein eigenes finanzielles System entwickeln, um den Umbau zu finanzieren eine bürokratische Belastung des Handels wolle man aber verhindern, heißt es. | Tax as the wrong instrument: Other sources of funding possible | Dagegen wird nicht genau klar, wie der nötige artgerechte Umbau der Tierhaltung finanziert werden soll, den die Koalition vorantreiben will. Die FDP war gegen eine Steuererhöhung, etwa auf Fleischprodukte, mit der der Umbau möglich gewesen wäre. Stattdessen sollen nun die »Marktteilnehmer« ein eigenes finanzielles System entwickeln, um den Umbau zu finanzieren. Eine bürokratische Belastung des Handels wolle man aber verhindern, heißt es. |
| WELT_2019-08-08_Tierwohl und Klimaschutz - Union, SPD und Gruen | Tierschutz sei im Grundgesetz verankert und müsse durch "ordnungsrechtliche Maßnahmen und zielgerichtete Förderpolitik" durchgesetzt werden. "Da haben Steuern nichts zu suchen." | Tax as the wrong instrument: Regulations and control of agricultural practices needed | Linke, FDP und AfD gegen Steuer Gegen eine Verteuerung von Fleisch spricht sich hingegen die Linke-Politikerin Kirsten Tackmann aus. "Ich halte den Ansatz für falsch, Tierschutz über eine Fleischsteuer regeln zu wollen, die ja auch sozial Benachteiligte zahlen müssten", sagte Tackmann. Tierschutz sei im Grundgesetz verankert und müsse durch "ordnungsrechtliche Maßnahmen und zielgerichtete Förderpolitik" durchgesetzt werden. "Da haben Steuern nichts zu suchen." |
| AH_2020-02-12_Breite Zustimmung zum Borchert-Plan fuer die Tier | Besser wären aus seiner Sicht eine Umschichtung bestehender Subventionen, ein staatliches Tierwohllabel und höhere gesetzliche Standards, die auch zu höheren Fleischpreisen für Verbraucher führen müssten. | Tax as the wrong instrument: Subsidies need to be reformed | Tier- und Verbraucherschützer gespalten [...] Eine Außenseiterrolle nahm die Verbraucherzentrale Bundesverband (vzbv) ein. Der vzbv begrüßte zwar die Empfehlungen, beendete aber dennoch seine Mitarbeit im Kompetenznetzwerk. Zur Begründung führte vzbv-Vorstand Klaus Müller den Vorschlag zur Einführung einer Tierwohlabgabe an, um die Umbaukosten zu finanzieren. Besser wären aus seiner Sicht eine Umschichtung bestehender Subventionen, ein staatliches Tierwohllabel und höhere gesetzliche Standards, die auch zu höheren Fleischpreisen für Verbraucher führen müssten. |

# 5. Example of extracted quotes for the same frame

| Article | Frame | Manual quote | LLM quote |
| --- | --- | --- | --- |
| AFZ_2020-09-09_Fleischkonsum soll teurer werden | Tax as causing unintended disruption (market/industry/farmers): Tax as hurting better products disproportionately | Durch eine mengenbezogene Abgabe wie beispielsweise eine Verbrauchssteuer würde eine höhere Besteuerung von besonders nachhaltig erzeugten (und damit besonders teuren) Produkten, wie etwa denen des ökologischen Landbaus, verhindert. | Würde besonders nachhaltig erzeugtes Fleisch dadurch noch teurer werden? Durch eine mengenbezogene Abgabe wie beispielsweise eine Verbrauchssteuer würde eine höhere Besteuerung von besonders nachhaltig erzeugten (und damit besonders teuren) Produkten, wie etwa denen des ökologischen Landbaus, verhindert. |
| FAZ_2019-08-13_Wer Fleisch isst, muss kein schlechtes Gewissen | Tax as hurting consumers: Tax as amplifying inequalities | Es würde sich nichts Wesentliches verändern – ich glaube, das werden nur Menschen merken, die sowieso schon jeden Euro zwei Mal umdrehen müssen. | Es würde sich nichts Wesentliches verändern - ich glaube, das werden nur Menschen merken, die sowieso schon jeden Euro zwei Mal umdrehen müssen. Und die kaufen selten beim Metzger ein. |
| FW_2020-05-01_Interview - Langfristig wird das Geld nicht reich | Tax as hurting consumers: Tax as amplifying inequalities | Ist eine Sonderabgabe Tierwohl nicht unsozial? Fischer: Eine mengenbasierte Sonderabgabe ist einerseits pauschal und andererseits einkommensunabhängig. Dadurch werden Preiseinstiegsprodukte und Haushalte mit niedrigem Einkommen stärker belastet. Ist das gerecht? | Eine mengenbasierte Sonderabgabe ist einerseits pauschal und andererseits einkommensunabhängig. Dadurch werden Preiseinstiegsprodukte und Haushalte mit niedrigem Einkommen stärker belastet. Ist das gerecht? |
| AFZ_2020-09-09_Fleischkonsum soll teurer werden | Tax as causing unintended disruption (market/industry/farmers): Tax as hurting better products disproportionately | Durch eine mengenbezogene Abgabe wie beispielsweise eine Verbrauchssteuer würde eine höhere Besteuerung von besonders nachhaltig erzeugten (und damit besonders teuren) Produkten, wie etwa denen des ökologischen Landbaus, verhindert. | Würde besonders nachhaltig erzeugtes Fleisch dadurch noch teurer werden? Durch eine mengenbezogene Abgabe wie beispielsweise eine Verbrauchssteuer würde eine höhere Besteuerung von besonders nachhaltig erzeugten (und damit besonders teuren) Produkten, wie etwa denen des ökologischen Landbaus, verhindert. |

# References

1. Leibinger A, Moerschel K, Hagge A, Hawkins B, Fesenfeld L, von Philipsborn P: **The German media debate on taxing meat and other animal-based foods: a framing analysis [Manuscript submitted for publication]**. *Chair of Public Health and Health Services Research, Faculty of Medicine, LMU Munich, Germany*.
